# Comparison between non-vitamin K oral antagonist versus warfarin in atrial fibrillation with and without valvular heart disease: a systematic review and meta-analysis

**DOI:** 10.1186/s43044-024-00535-w

**Published:** 2024-08-09

**Authors:** Arga Setyo Adji, Bryan Gervais de Liyis

**Affiliations:** 1 Faculty of Medicine, Hang Tuah University, Surabaya, East Java Indonesia; 2 Faculty of Medicine, Udayana University, Denpasar, Bali Indonesia

**Keywords:** Atrial fibrillation, Valvular heart disease, Vitamin K, Warfarin

## Abstract

**Background:**

Atrial fibrillation (AF) poses a significant stroke risk in heart disease patients. This systematic review aims to evaluate the efficacy and safety of non-vitamin K oral antagonists (NOACs) versus vitamin K antagonists (VKAs) in AF patients with and without any valvular heart disease (VHD/N-VHD).

**Methods:**

A systematic search was conducted on PubMed, Scopus, and Google Scholar up to March 3, 2022. Efficacy and safety parameters were analyzed.

**Results:**

A total of 85,423 subjects from 10 studies were included in this meta-analysis. NOACs and VKAs showed similar effects on ischemic stroke in AF patients with VHD/N-VHD (RR 0.97; 95% CI 0.72–1.30; *p* = 0.83) and also on systemic embolic events (RR 1.02; 95% CI 0.83–1.25; *p* = 0.86). Similar effects were seen in VHD and N-VHD subgroups. Both treatments had similar effects on myocardial infarction in AF patients with VHD/N-VHD (RR 0.79; 95% CI 0.49–1.26; *p* = 0.32), VHD (RR 0.78; 95% CI 0.59–1.02; *p* = 0.07), and N-VHD subgroups (RR 0.82; 95% CI 0.30–2.21; *p* = 0.69). NOACs reduced the risk of intracranial bleeding in AF VHD/N-VHD (RR 0.64; 95% CI 0.54–0.77; *p* < 0.0001), VHD (RR 0.59; 95% CI 0.42–0.82; *p* = 0.002), and N-VHD subgroups (RR 0.70; 95% CI 0.57–0.85; *p* = 0.0003). Additionally, NOACs reduced the risk of gastrointestinal bleeding in AF VHD/N-VHD (RR 0.80; 95% CI 0.66–0.96; *p* = 0.02), specifically in the VHD subgroup (RR 0.69; 95% CI 0.54–0.89; *p* = 0.004). Moreover, NOACs were associated with a decreased risk for minor and non-fatal bleeding in AF patients with VHD/N-VHD (RR 0.86; 95% CI 0.75–0.99; *p* = 0.04).

**Conclusion:**

NOACs are effective and safe for ischemic stroke, systemic embolic events, myocardial infarction, intracranial bleeding, and gastrointestinal bleeding in AF patients with VHD/N-VHD.

## Background

Atrial fibrillation (AF) is a common kind of heart arrhythmia. AF develops when tachyarrhythmia, an irregular electrical action in the heart’s atrium, starts fibrillation. A variety of symptoms, including but not limited to chest pain, palpitations, fast heartbeat, difficulty breathing, nausea, vertigo, profuse perspiration, and weakness, can accompany atrial fibrillation (AF) [1–3]. In establishing the diagnosis of AF, the electrocardiogram (ECG) examinations are crucial in confirming the diagnosis of atrial fibrillation (AF). The electrocardiogram (ECG) reveals a complex narrow pattern that is “irregularly irregular” and does not contain any discernible p waves. With ventricular rates ranging from 80 to 180 beats per minute, fibrillar waves might or might not be visible [4]. Age, hypertension, preexisting cardiac conditions, congenital heart defects, and alcohol consumption are all risk factors for atrial fibrillation [1–3].

Management of cardioversion in AF can be achieved through pharmacological or electrical means. Intravenous amiodarone is the only available antiarrhythmic medication in Egypt for acute AF cardioversion, and it can take between 6 and 16 h to be effective [5]. Identifying clinical factors that predict early successful cardioversion can help in deciding whether to use pharmacological or electrical cardioversion, potentially reducing hospital stay and associated costs. For instance, the use of antiarrhythmic agents such as encainide, digoxin, and amiodarone has been associated with an increased propensity for rhythm conversion in patients undergoing cardioversion [6, 7].

Atrial fibrillation (AF) in valvular heart disease (VHD) remains a concern. According to the 2023 ACC/AHA/ACCP/HRS guidelines, VHD encompasses any dysfunction or abnormality in one or more of the heart’s four valves: the aortic, mitral, pulmonary, and tricuspid valves. These issues can manifest as stenosis (a narrowing that restricts blood flow) or regurgitation (backward leakage due to improper valve closure). If not properly managed, VHD can result in significant morbidity and mortality [8]. Based on the 2021 ESC/EACTS guidelines, the management of valvular heart disease (VHD) varies significantly depending on the type and severity of the valve dysfunction. For mild cases, conservative management with regular monitoring is typically recommended; lifestyle modifications and medical management are needed; contributing conditions like hypertension or heart failure may be recommended. In moderate cases, medical therapy is often employed to manage symptoms and prevent complications, with interventions considered if symptoms worsen. For severe cases, more aggressive interventions such as valve repair or replacement are necessary; options include valve repair or replacement, which can be done via traditional open-heart surgery or minimally invasive techniques like transcatheter aortic valve replacement (TAVR) or mitral valve repair. The choice between surgical and transcatheter techniques depends on various factors, including patient-specific characteristics, procedural risks, and the expertise of the heart team. The guidelines emphasize individualized treatment plans and the importance of a multidisciplinary approach to optimize patient outcomes [9].

In cases of cardiac disease, AF is the primary determinant of stroke risk [10]. Because of the irregular heartbeat, the patient’s blood flow becomes turbulent, increasing the risk of thrombus formation and, in the worst-case scenario, a stroke [1–3]. The occurrence of AF has been on the rise worldwide. The prevalence of AF seems to increase with age. By the year 2050, the number of individuals diagnosed with atrial fibrillation will probably have increased by a factor of two or three. Atrial fibrillation affects more than 9 percent of individuals 75 and older, despite a global prevalence of less than 1%. Atrial fibrillation (AF) is 22% more common in people aged 80 and up [11, 12]. The incidence of atrial fibrillation (AF) is higher in developed nations compared to less developed ones, and it affects males more often than women [13].

Anticoagulants, rate-controlling medications, rhythm, cardioversion, ablation, and other cardiac operations can lower the risk of stroke in persons with atrial fibrillation (AF) [1–3]. Vitamin K antagonists (VKAs) and non-vitamin K oral anticoagulants (NOACs) are necessary for the anticoagulation of atrial fibrillation (AF), which is intended to prevent stroke. The use of VKAs persisted in falling after the four NOACs—dabigatran, rivaroxaban, apixaban, and edoxaban—were agreed upon [14]. When comparing warfarin with NOAC, the only group that warrants an exception are patients who have mechanical heart valves and moderate to severe mitral stenosis (MS) [15, 16]. When compared to VKA, NOAC is just as effective and safer in reducing the risk of stroke or bleeding, if not safer [17, 18].

A decreased incidence of blood loss events equivalent to VKA was associated with the combined therapy strategy involving NOAC. Triple antithrombotic therapy (TAT) considerably decreased risk when added to dual antithrombotic therapy (DAT). Compared to TAT with VKA and dual antiplatelet therapy (DAPT), DAT with NOAC and single antiplatelet therapy (SAPT) reduced the relative risk (RR 0.63; 95% CI 0.50–0.80) by 37%. Neither VKA nor treatment approaches involving a combination of NOAC were associated with a lower risk of stroke or death in prior research [19]. Thus, in order to prevent AF patients from developing valvular heart disease (VHD), a comprehensive evaluation of therapeutic approaches is required, including the use of NOAC or VKA for stroke prevention.

## Methods

This systematic review was conducted on the PRISMA protocol [20]. The registration number for this review procedure is CRD42022357998, and it is part of the International Prospective Register of Systematic Reviews (PROSPERO).

### Eligibility criteria

For the sake of this meta-analysis, we strictly adhered to inclusion and exclusion criteria. Valvular AF is defined as AF occurring in patients with mitral stenosis (MS), mitral regurgitation (MR), aortic stenosis (AS), aortic regurgitation (AR), mechanical heart valves, or those who have undergone valve replacement. These conditions are considered inclusion criteria for valvular AF. In contrast, non-valvular AF is defined as AF not caused by a heart valve issue. Studies included in this meta-analysis specifically addressed either valvular or non-valvular AF. We excluded studies that did not focus on individuals with AF related to valvular or non-valvular heart disease (N-VHD). The following criteria were also considered: (1) efficacy in the form of an ischemic stroke; (2) safety in the form of myocardial infarction, intracranial bleeding, or gastrointestinal bleeding; (3) the use of a cross-sectional, cohort, or case–control study design; and (4) the research had to have been written in English. On the flip side, we did not include trials that did not compare NOACs to warfarin or that did not have appropriate outcome measures. Additionally, in order to guarantee that the results were applicable to the intended group of AF patients, data from non-human studies were also omitted from the study.

### Search strategy and selection of studies

A number of databases were searched in order to find pertinent subjects up until March 2022 including PubMed, ScienceDirect, and Google Scholar. Formula search terms included “anticoagulant,” “NOAC,” “novel oral anticoagulant,” “VKA,” “vitamin K antagonist,” “atrial fibrillation,” “stroke,” “intracranial hemorrhage,” “vascular heart disease,” and “non-vascular heart disease.” Boolean “AND” and “OR” were also utilized. By perusing the publications’ citations, we were able to uncover additional studies that were comparable or pertinent.

### Data extraction

Designated investigators (A.S.A. and B.G.L.) painstakingly extracted relevant data using a predetermined data extraction form after relevant studies were selected. Study features including author, publication year, and study design were among the many aspects covered by the extracted data. Additionally, participant demographics, interventions administered (including type and dosage of NOACs or warfarin), and reported outcome measures were systematically recorded. To maintain the integrity and precision of the data extraction process, a thorough cross-checking procedure was implemented. Another investigator independently reviewed the extracted data to verify its accuracy and completeness, thereby mitigating the risk of errors or omissions. This stringent validation process ensured the reliability and robustness of the extracted data for subsequent analysis.

### Quality assessment

The Newcastle–Ottawa Scale (NOS), developed for use in non-randomized research designs, was used to conduct additional evaluations of each of the publications that were included in the list. There are three points for exposure determination, four for patient selection, and two for group comparability in the NOS. Total scores could range from 0 (very bad) to 9 (excellent). The Cochrane risk-of-bias instrument for randomized trials, second edition (RoB 2), was utilized to assess RCT publications for potential bias in a more comprehensive manner. The RoB2 tools automatically do all computations and evaluations in accordance with five critical areas [21, 22]. The quality evaluation was carried out by two researchers (A.S.A.). If any conflicts arise during this evaluation period, the researchers will work together to find a solution.

### Outcome measure

The analysis considered several outcome measures, encompassing efficacy and safety. Efficacy was evaluated in terms of ischemic stroke incidence, while safety endpoints included myocardial infarction, intracranial bleeding, and gastrointestinal bleeding.

### Data synthesis and statistical analysis

Every outcome measure in this study had its own pooled risk ratio (RR) and 95% CI, which were determined by a meta-analysis. To determine how heterogeneous the included studies were, we used the *I*^2^ statistic. Patients undergoing atrial fibrillation can be categorized into subgroups according to the presence or absence of ventricular HF and non-ventricular HF. The results will also be subjected to sensitivity analysis to ensure their robustness. Statistical significance was defined as a *p* value less than 0.05. With the help of Review Manager 5.4, we ran the statistical analysis [23].

## Results

### Study selection process and quality assessment

**Fig. 1:**
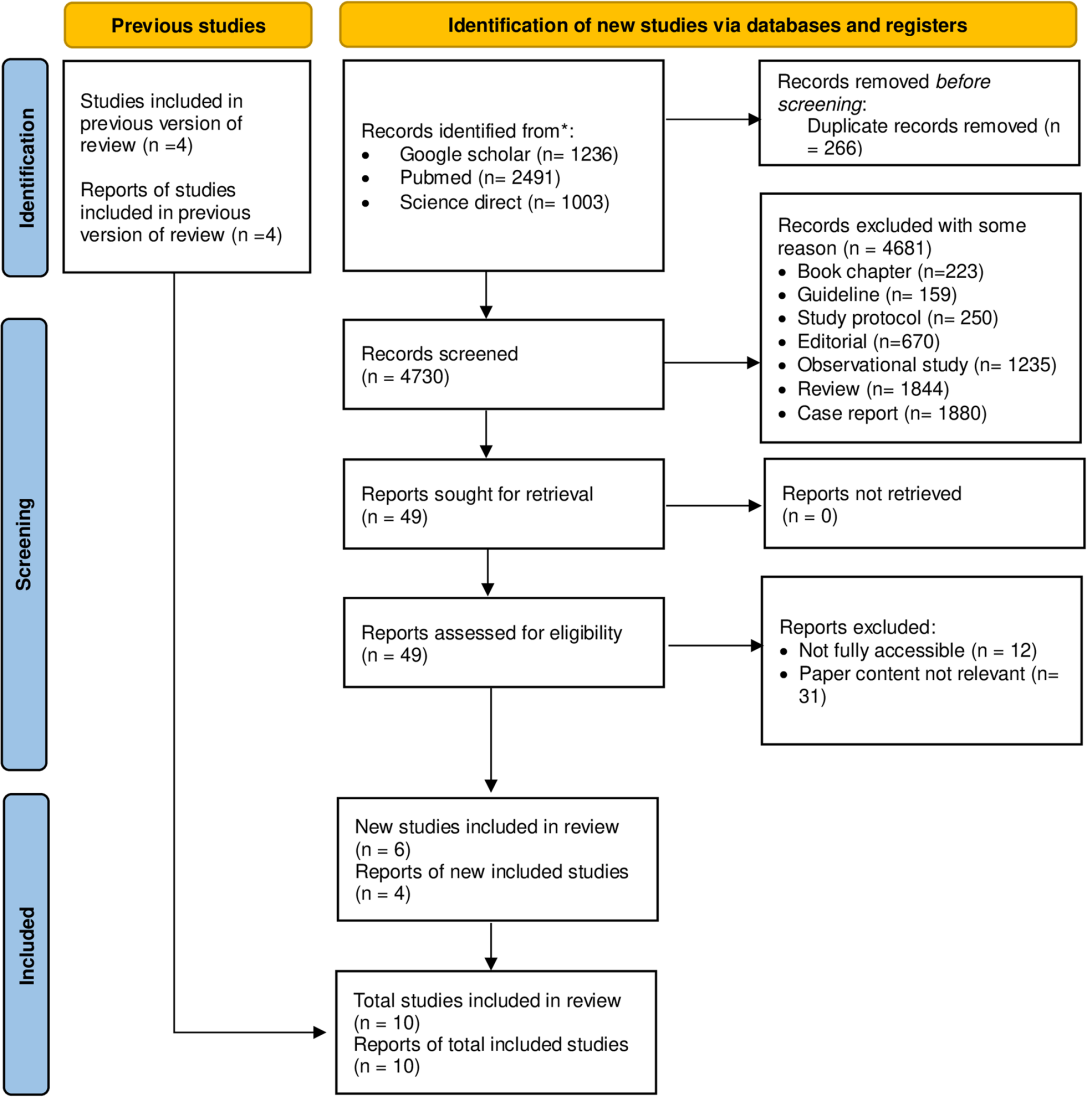
PRISMA flow diagram of the study selection process

After searching online sources such as PubMed, ScienceDirect, and Google Scholar, the authors discovered four studies out of 4730. After being rejected for reasons that are yet unclear, 49 studies were kept. Six studies were deemed eligible for assessment following paper content and paper-accessible screening. The total report of 6 new included studies, and the previous is 10. Here is a flowchart that summarizes the complete literature search process according to the PRISMA Guideline: (Fig. 1).

### Study characteristics

Table 1 shows the outcomes of interest associated with AF with or without VHD, as determined by a total of ten investigations [24–33], involving 85,423 individuals. With four studies each, Asia, the United States, and Europe accounted for the bulk of the participants. Three months to three and a half years was the range of the follow-up duration.

**Table 1:** Data characteristics

No	Author, year	Study design	Location (country)	Population		Total population	VHD					N-VHD	Mean age ± S.D (year)*
				NOACs	VKAs		AS	AR	TR	MS	MR		
1	Breithardt et al., 2014	RCT	Multicenter (USA)	5318	8853	14,171	215	486	NA	NA	1756	12,179	73 (65, 78)
2	Avezum et al., 2015	RCT	Multicenter (Brazil)	2438	2370	4808	384	887	2124	131	3526	4808	71 (64, 77)
3	Ezekowitz et al., 2016	RCT	Multicenter (USA)	12,090	6022	18,112	471	817	1179	193	3101	14,162	74.00 (68.00, 78.00) for dabigatran 110 mg in VHD patients, 74.00 (67.00, 79.00) for dabigatran 150 mg in VHD patients, and 74.00 (68.00, 79.00) for Warfarin in VHD patients
4	Caterina et al., 2017	RCT	Multicenter (Italy)	N.A	N.A	21,046	165	369	NA	NA	2250	18,222	71.8 ± 9.4 (VHD); 70.4 ± 9.4 (N-VHD)
5	Kanai et al., 2017	Cohort	Japan	70	31	101	NA	NA	NA	NA	NA	101	81 (73–87) (NOAC); 81 (77–87) (VKA Warfarin)
6	Moon et al., 2019	Cohort	South Korea	2792	2371	5163	3214			92	2423	NA	71.2 ± 9.9 for Warfarin and 71.2 ± 8.4 for NOAC
7	Kim et al., 2020	Cohort	Multicenter (South Korea)	795	3158	3953	NA	NA	NA	NA	NA	3953	71.7 ± 8.4
8	Li et al., 2021	Cohort	Taiwan	5833	5833	11,666	11,666					NA	76.48 ± 10.38 for NOAC and 75.68 ± 10.76
9	Melgaard et al., 2021	Cohort	Denmark	1369	2357	3726	3726	NA	NA	NA	NA	NA	79 (73–85) for warfarin and 82 (75–88)
10	Strange et al., 2021	Cohort	Denmark	1115	1562	2677	1653	625	NA	NA	739	NA	77.0 (70.0–83.0) for VKA, 80.0 (71.0–87.0) for Rivaroxaban, and 81.00 (74.0–87.8) for Apixaban

^*^Reported in median values

*AS* Aortic Stenosis, *AR* Aortic Regurgitation, *MS* Mitral Stenosis, *MR* Mitral Regurgitation, *N-VHD* Non-Valvular Heart Disease, *TR* Tricuspid Regurgitation, *VHD* Valvular Heart Disease

### Risk of bias

Table 2 shows the categorization of the ten included studies according to the methodologies used to determine their risk of bias. Using the Newcastle–Ottawa Scale (NOS), six studies were evaluated; five of them were deemed to have good quality, while one was deemed to have fair/moderate quality. All of the studies that were considered by RoB to have a low risk of bias, with the exception of four others, indicate that the studies that were included are of good quality.

**Table 2:** Study outcome

No	Author, year	Intervention drug	Comparator	Treatment duration	Major outcomes	Risk of bias / quality of study
1	Breithardt et al., 2014	Rivaroxaban	Warfarin	840 days	In both individuals with and without SVD, rivaroxaban showed a comparable risk of stroke or systemic embolism to that of warfarin. Patients were randomized to fixe dose rivaroxaban 20 mg once daily; 15 mg daily for individuals with moderate renal impairment (creatinine clearance of 30–49 mL/min)	Low
2	Avezum et al., 2015	Apixaban	Warfarin	30 months	There was no evidence suggesting that apixaban provided greater benefits than warfarin in reducing the occurrence of strokes in patients with VHD or N-VHD. The dose of apixaban were randomly, 5 mg twice daily and 2.5 mg (by ≥ 2 of the following: age ≥ 80 years, body weight ≤ 60 kg, or serum creatinine ≥ 133 μmol/L (1.5 mg/dL)	Low
3	Ezekowitz et al., 2016	Dabigatran	Warfarin	36 months	In individuals with either VHD or N-VHD, the occurrence of strokes was less frequent among those treated with dabigatran 150 mg compared to those on warfarin. However, the rates of stroke were similar between patients on warfarin and those taking dabigatran 110 mg, irrespective of whether they had VHD or N-VHD	Low
4	Caterina et al., 2017	Edoxaban	Warfarin	3.5 years	In individuals without VHD, it seems that edoxaban showed a more favorable outcome in terms of lowering overall mortality and the combined occurrence of death or severe stroke compared to warfarin	Low
5	Kanai et al., 2017	NOAC	VKA	4 years	Using NOACs for secondary prevention post-stroke could potentially offer greater benefits compared to VKAs, as it may decrease the volume of recurrent infarcts	Good
6	Moon et al., 2019	NOAC	Warfarin	1.4 years	When comparing NOACs with warfarin, it was found that NOACs were associated with decreased risks of ischemic stroke, major bleeding events, overall mortality, and a combined outcome	Fair
7	Kim et al., 2020	VKA	Dabigatran, Apixaban, Rivaroxaban, Edoxaban	12 months	In both groups of patients continuing their medication and those just starting treatment, the rate of discontinuation was notably lower with NOACs compared to VKAs	Good
8	Li et al., 2021	NOAC	Warfarin	6.0 months for NOAC and 7.7 months for warfarin	In individuals with AF and VHD, NOACs demonstrated a similar risk of ischemic stroke and bleeding when compared to warfarin	Good
9	Melgaard et al., 2021	Warfarin	NOAC	3 years	In individuals with atrial fibrillation and aortic stenosis, NOACs showed a higher likelihood of thromboembolism but a reduced risk of severe bleeding compared to warfarin	Good
10	Strange et al., 2021	VKA	Rivaroxaban and apixaban	2 years	Patients diagnosed with AF and VHD who were administered VKAs versus Factor Xa inhibitors did not show any notable variances in the likelihoods of experiencing all-cause mortality, stroke, or hemorrhage. Rivaroxaban 20 mg once daily and apixaban 5 mg twice daily	Fair

### Efficacy of non-vitamin k oral antagonists versus warfarin

Table 3 offers a concise evaluation of the safety and effectiveness of NOACs compared to warfarin in treating atrial fibrillation (AF) in individuals with and without valvular heart disease (VHD).

**Table 3:** Summary of results

End Point	AF with Valvular Heart Disease and Non-Valvular Heart Disease		
	VHD N:WRR (95% CI)	N-VHD N:WRR (95% CI)	p value
Efficacy			
Ischemic stroke	0.88 (0.75–1.04)	1.13 (0.64–1.99)	0.83
Systemic embolic events	1.09 (0.78–1.53)	0.94 (0.83–1.07)	0.86
Safety			
Myocardial infraction	0.78 (0.59–1.02)	0.82 (0.30–2.21)	0.32
Intracranial bleeding	0.59 (0.42–0.82)	0.70 (0.57–0.85)	0.00001^*^
Gastrointestinal bleeding	0.69 (0.54–0.89)	0.96 (0.69–1.34)	0.02^*^
Minor and non-fatal bleeding	0.93 (0.81–1.07)	0.82(0.66–0.99)	0.04*

*CI* Confidence interval, *N* NOAC (Non-Vitamin K Antagonist), *N-VHD* Non-Valvular Heart Disease, *RR* Risk ratio, *VHD* Valvular Heart Disease, *W* Warfarin

#### Ischemic stroke

**Fig. 2:**
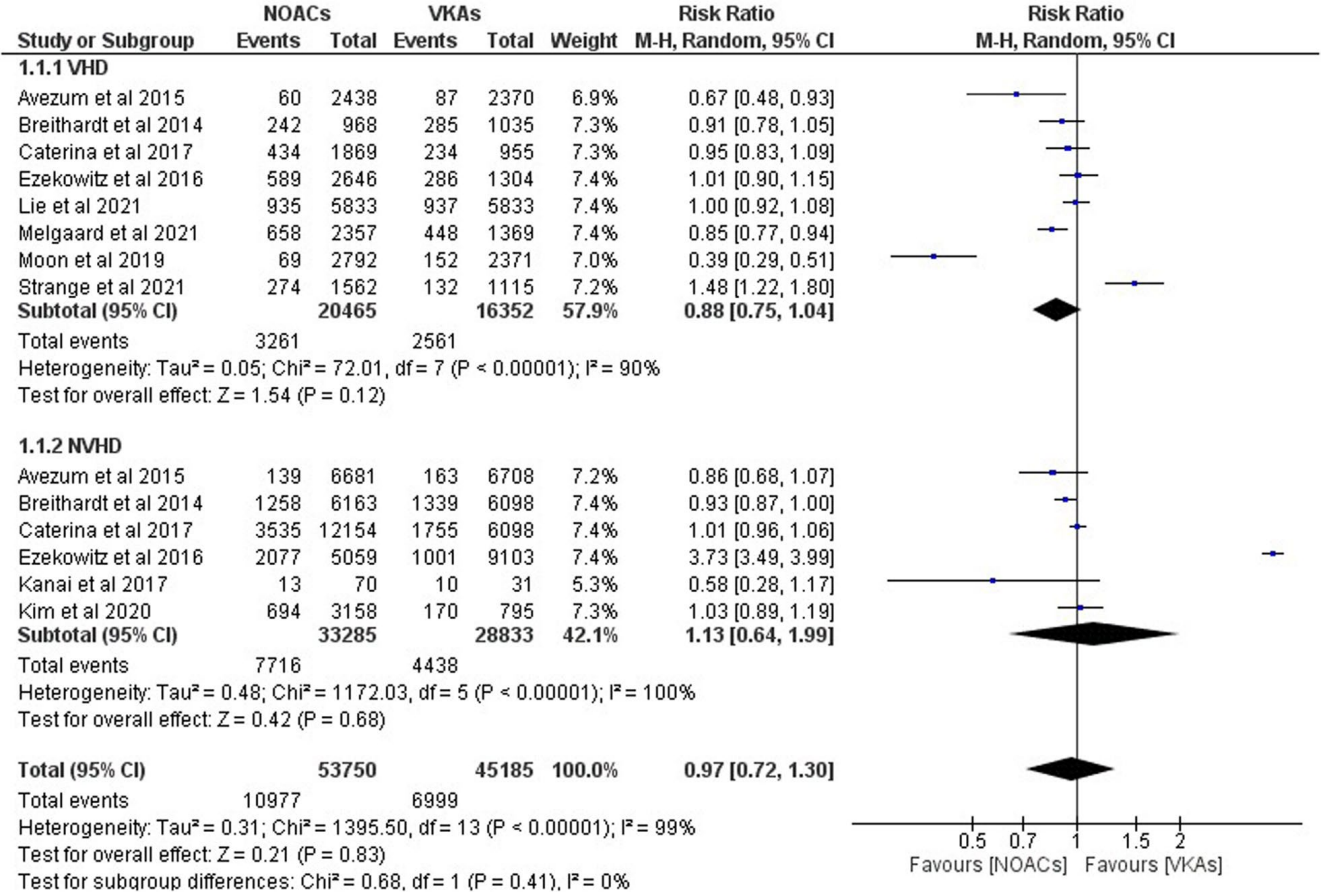
Forest plot of NOACs vs VKAs in ischemic stroke

In Fig. 2, a total of 10 studies represents the total number of participants in each treatment group across all the included studies. There were 53,750 participants in the studies treated with NOACs and 45,185 participants treated with VKAs. We found no statistical difference between NOACs and VKAs in relation to ischemic stroke in VHD “(RR 0.88; 95% CI 0.75–1.04; *p* = 0.12; *I*^2^ = 90%)” and N-VHD “(RR 1.13; 95% CI 0.64–1.99; *p* = 0.68; I^2^ = 100%)” when we pooled our data. When it came to ischemic stroke, NOACs and VKAs had comparable effects in AF patients with VHD/N-VHD “(RR 0.97; 95% CI 0.72–1.30; *p* = 0.83; *I*^2^ = 99%).”

#### Systemic embolic events (SEE)

**Fig. 3:**
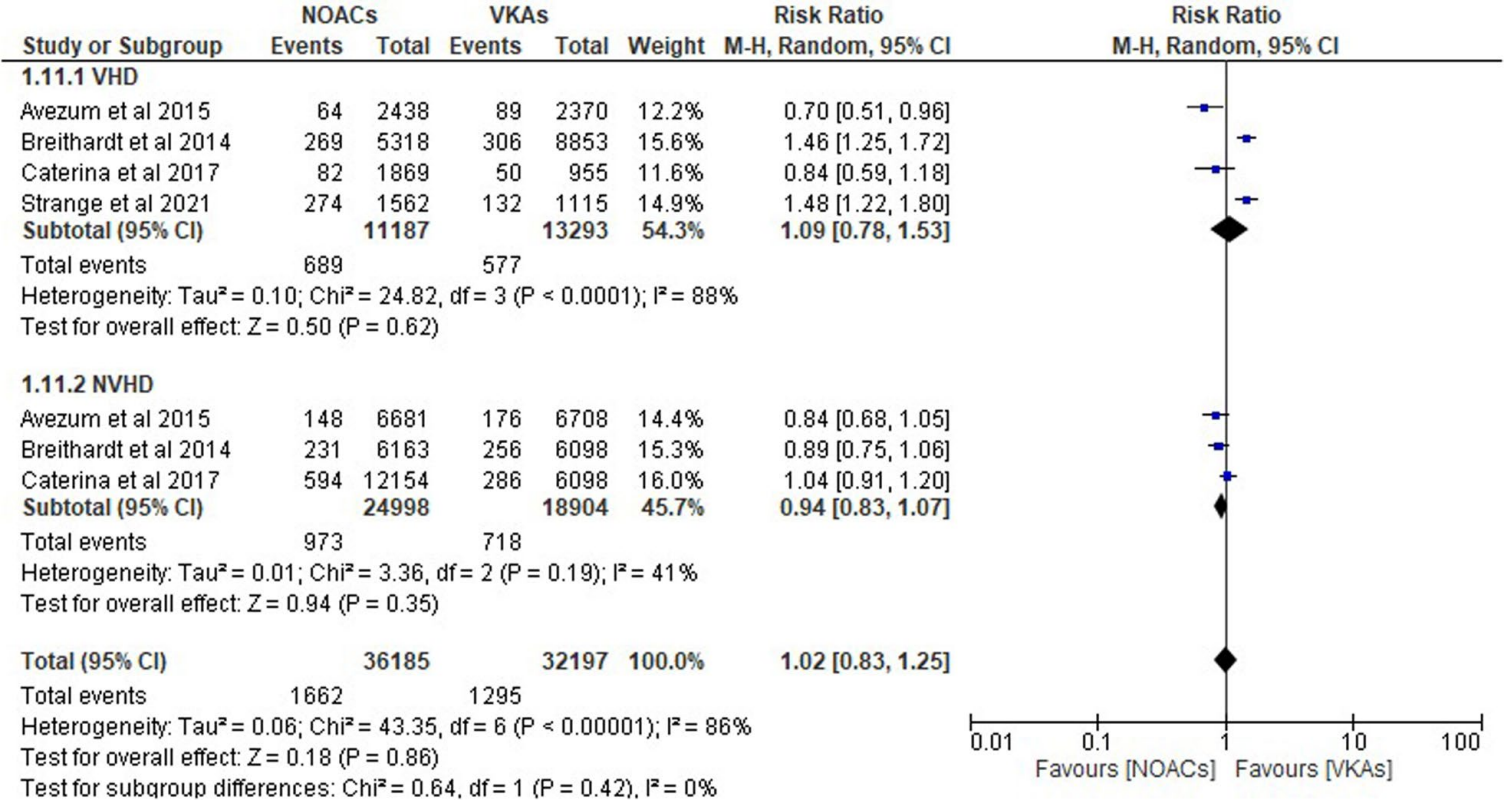
Forest plot of NOACs vs VKAs in systemic embolic events

In Fig. 3, the comparison of systemic embolic events between non-vitamin K oral anticoagulants (NOACs) and warfarin in atrial fibrillation (AF) patients with and without valvular heart disease (VHD) shows varied results. For patients with VHD, the relative risk (RR) of systemic embolic events was 1.09 (95% CI 0.78–1.53), indicating no significant difference between NOACs and warfarin. For patients without VHD (N-VHD), the RR was 0.94 (95% CI 0.83–1.07), also suggesting no significant difference between the two treatments. Overall, the pooled analysis found no statistical difference between NOACs and warfarin regarding systemic embolic events in AF patients regardless of the presence of VHD, with an RR of 0.86.

### Safety of non-vitamin k oral antagonists versus warfarin

#### Myocardial infraction

**Fig. 4:**
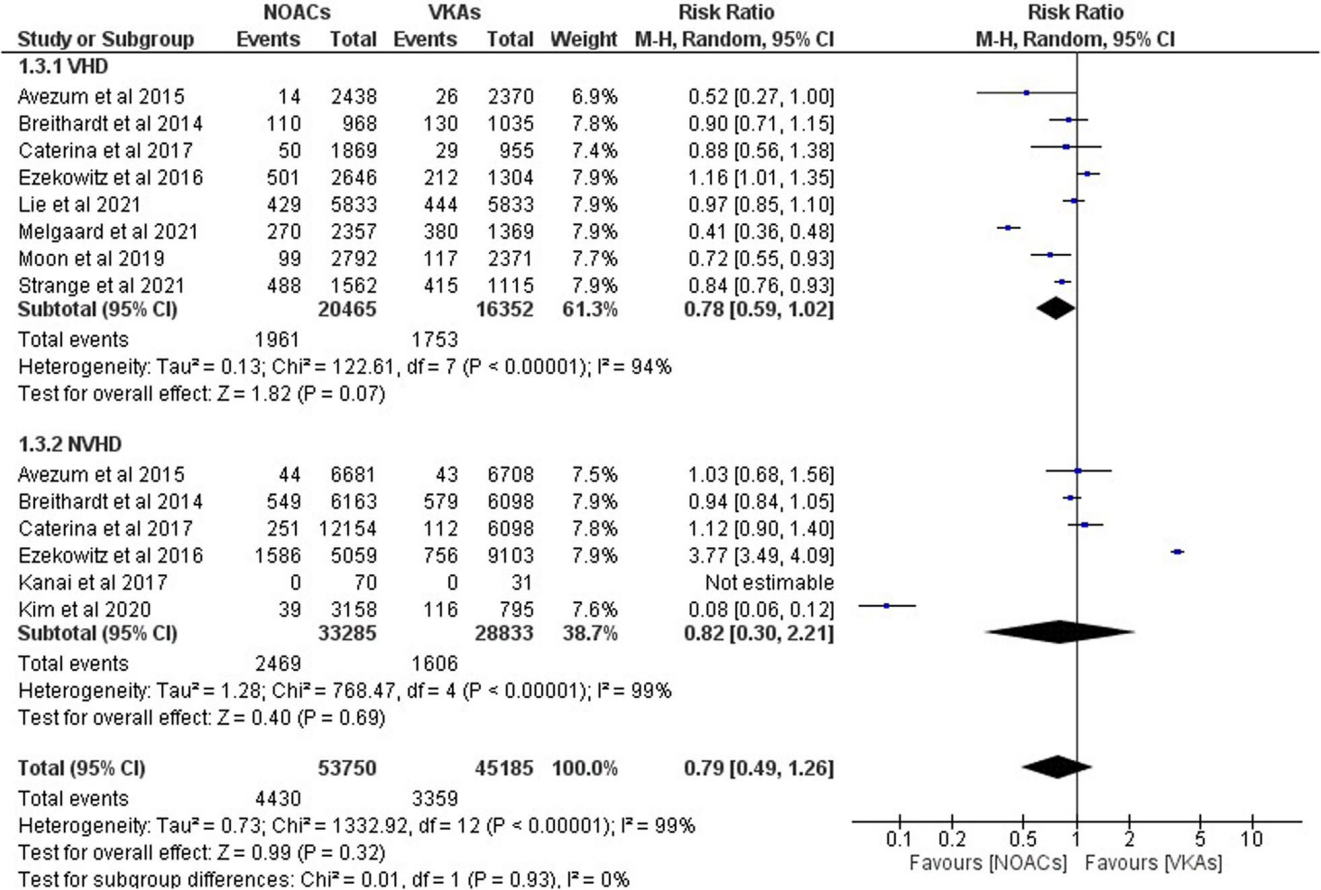
Forest plot of NOACs vs VKAs in myocardial infraction

Figure 4 shows the results of the ten studies that looked at the risk ratio of AF patients with and without VHD in MI and how NOACs compared to VKAs. In the VHD “(RR 0.78; 95% CI 0.59–1.02; *p* = 0.07; *I*^2^ = 94%)” and N-VHD “(RR 0.82; 95% CI 0.30–2.21; *p* = 0.69; *I*^2^ = 99%)” groups, our combined analysis did not find a statistically significant difference in the risk of MI between NOACs and VKAs. Comparing NOAC and VKA effects on MI in AF patients with VHD/N-VHD, we find that they are similar “(RR 0.79; 95% CI 0.49–1.26; *p* = 0.32; *I*^2^ = 99%).”

#### Intracranial bleeding

**Fig. 5:**
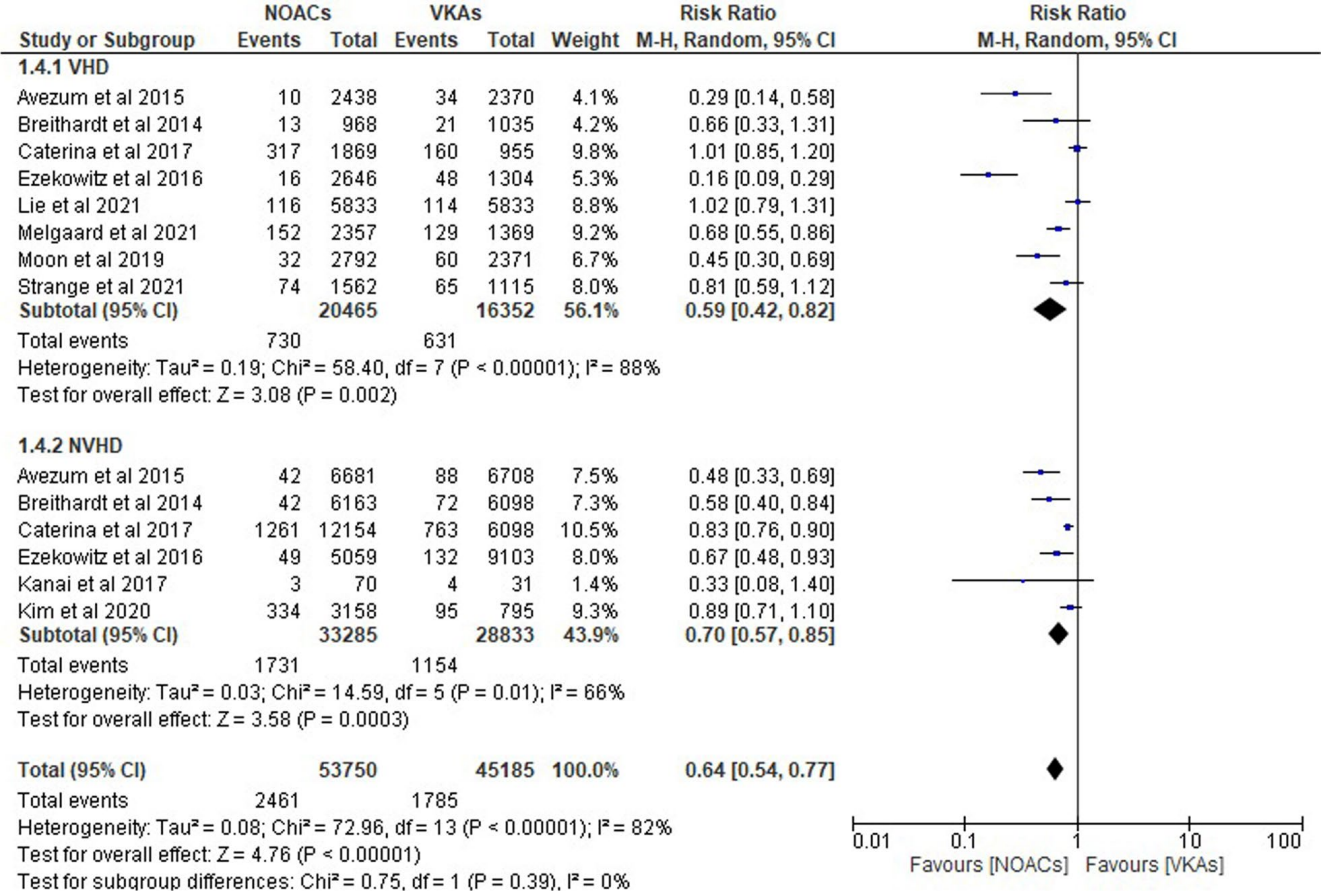
Forest plot of NOACs vs VKAs in intracranial bleeding

Figure 5 shows the results of 10 trials that demonstrated a decreased incidence of intracranial bleeding in patients with atrial fibrillation, regardless of whether they had valvular heart disease (VHD) or not, and in those who used NOACs instead of VKAs. We found that compared to VKAs, NOACs significantly reduce the incidence of intracranial hemorrhage in valvular heart disease patients by 41% in VHD “(RR 0.59; 95% CI 0.42–0.82; *p* = 0.002; *I*^2^ = 88%)” and by 30% in non-valvular heart disease patients “(RR 0.70; 95% CI 0.57–0.85; *p* = 0.0003; *I*^2^ = 66%).” The risk of cerebral hemorrhage was generally decreased by NOACs “(RR 0.64; 95% CI 0.54–0.77; *p* < 0.0001; *I*^2^ = 82%).”

#### Gastrointestinal bleeding

**Fig. 6:**
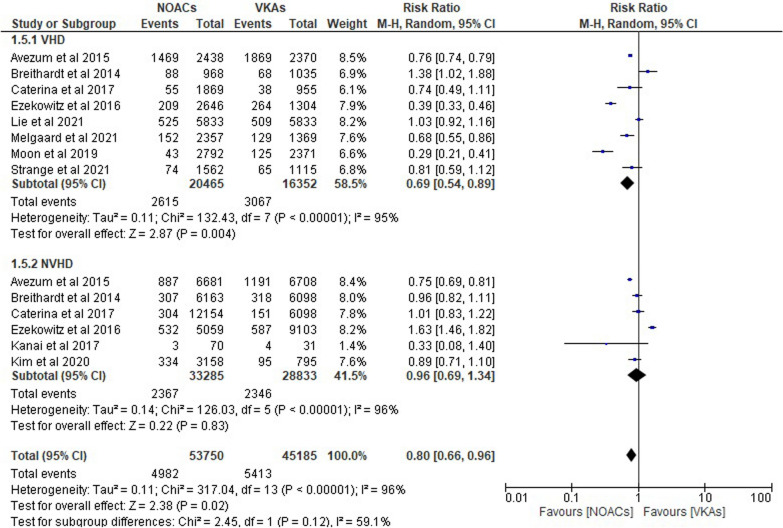
Forest plot of NOACs vs VKAs in gastrointestinal bleeding

In terms of the risk ratio of gastrointestinal bleeding in patients with AF and those without VHD, Fig. 6 displays the results of ten trials that compared NOACs and VKAs. When we combined the data from the studies, we found that NOACs were far more likely to cause gastrointestinal bleeding than VKAs. A 31% reduction in the incidence of cerebral hemorrhage was observed in patients with VHD when NOACs were used instead of VKAs “(RR 0.69; 95% CI 0.54–0.89; *p* = 0.004; *I*^2^ = 95%).” However, in patients without VHD, the opposite was true “(RR 0.96; 95% CI 0.69–1.34; *p* = 0.83; *I*^2^ = 96%).” Patients with atrial fibrillation who did not have ventricular fibrillation and who used NOACs had a reduced risk of gastrointestinal bleeding “(RR 0.80; 95% CI 0.66–0.96; *p* = 0.02; *I*^2^ = 96%).”

#### Minor and non-fatal bleeding

**Fig. 7:**
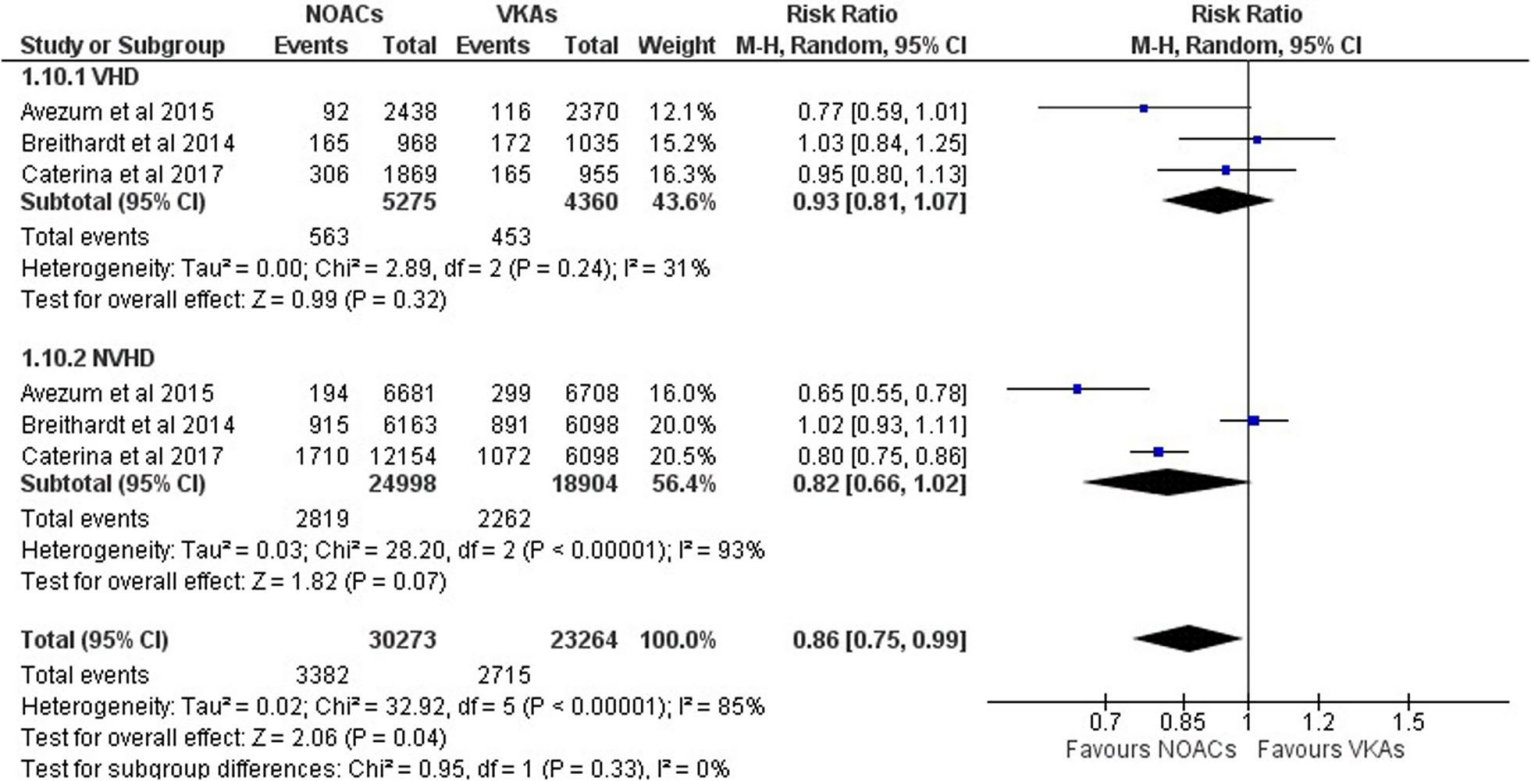
Forest plot of NOACs vs VKAs in minor and non-fatal bleeding

Figure 7 shows the results of the three studies that looked at the risk ratio of AF patients with and without VHD in minor and non-fatal bleeding and how NOACs compared to VKAs. In the VHD “(RR 0.93; 95% CI 0.81–1.07; *p* = 0.32; *I*^2^ = 31%)” and N-VHD “(RR 0.82; 95% CI 0.66–1.02; *p* = 0.07; *I*^2^ = 93%)” groups, our analyses did not find a statistically significant difference in the risk of minor and non-fatal bleeding between NOACs and VKAs. However, comparing NOAC and VKA effects in AF patients with VHD/N-VHD, we found that NOACs were associated with a decreased risk for minor and non-fatal bleeding “(RR 0.86; 95% CI 0.75–0.99; *p* = 0.04; *I*^2^ = 85%).”

**Fig. 8:**
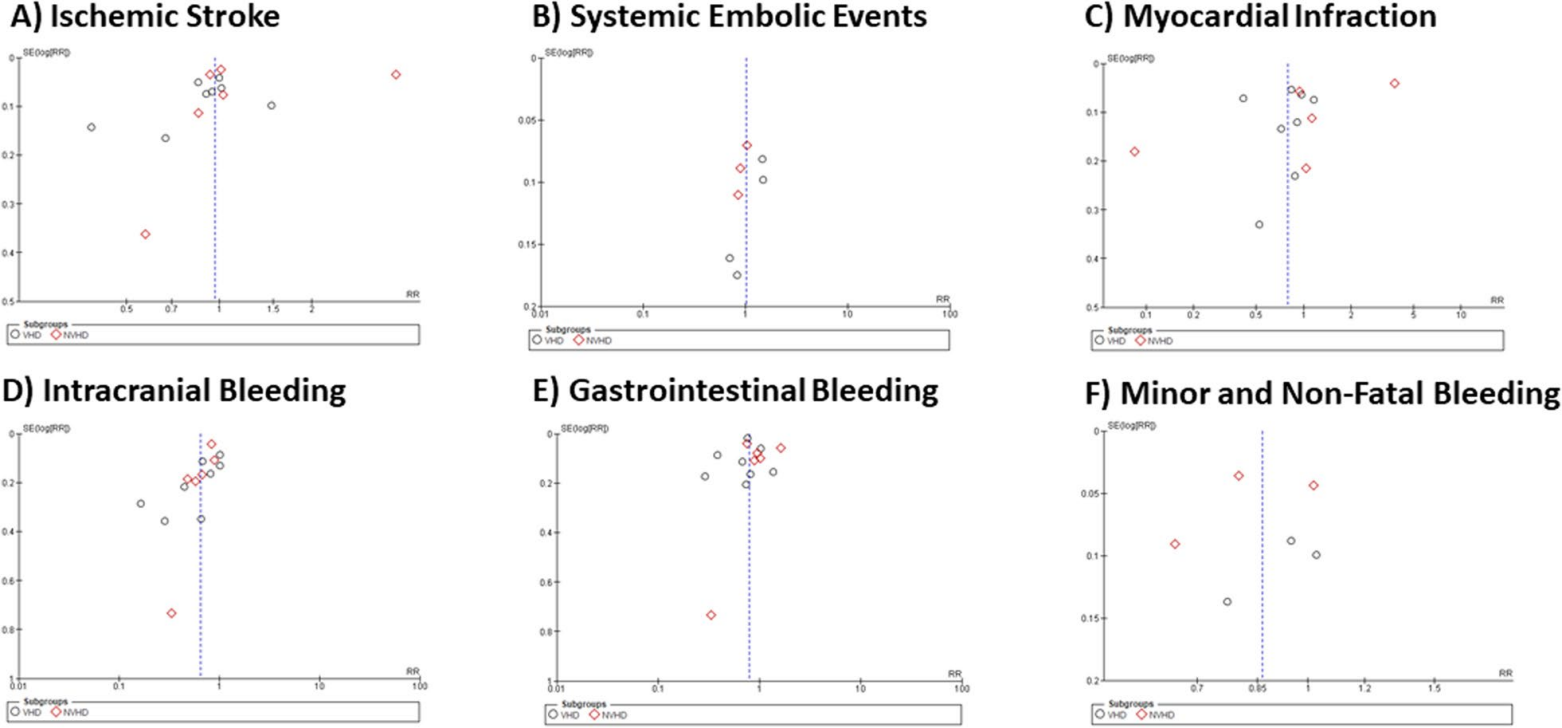
Funnel plot of publication bias for **A** Ischemic Stroke, **B** Systemic Embolic Events, **C** Myocardial Infraction, **D** Intracranial Bleeding, **E** Gastrointestinal Bleeding and **F** Minor and Non-Fatal Bleeding

The end points that were evaluated showed generally symmetrical funnel plots with estimable odds ratios (Fig. 8), suggesting that there was no major publication bias.

## Discussions

This meta-analysis assessed the safety and effectiveness of oral anticoagulants with and without vitamin K antagonists, drawing on data from 10 trials with 85,423 participants. The trials included warfarin, a drug that blocks the effects of vitamin K. Our research shows that NOACs greatly decrease the likelihood of bleeding in the brain and gastrointestinal system. This study shows that there is no statistical difference between NOACs and VKAs in relation to ischemic stroke, systemic embolic events (SEE), risk of MI, and minor and non-fatal bleeding. However, comparing NOAC and VKA effects in AF patients with VHD/N-VHD, we found that NOACs were associated with a decreased risk for minor and non-fatal bleeding. This result is in line with the previous study which reported that there is no significant difference between the NOACs compared to warfarin in terms of all-cause mortality and MI, but the risk of major bleeding events was found lower in patients receiving NOACs [34]. The results of the comparison between VKAs and NOACs may have different meanings and may differ significantly when analyzed separately for AF VHD and N-VHD. Combining these results may obscure potential differences as this study reported no significant difference was observed between DOACs and VKAs in AF regardless of AF etiology.

In this study, VHD classifications included aortic stenosis (AS), aortic regurgitation (AR), tricuspid regurgitation (TR), MS, and mitral regurgitation (MR). Among the included studies, the population receiving NOACs and warfarin for ischemic stroke and SEE was more frequent in AF patients with MR (22,250 patients). This study found no statistical difference between NOACs and VKAs in preventing ischemic stroke and SEE. According to Fanaroff et al., in patients with AF and MR, NOACs have been shown to be as effective as VKAs in reducing the risk of stroke and systemic embolism. Pivotal trials demonstrated that the effect of NOACs compared to VKAs on the primary efficacy endpoint of stroke and systemic embolism was similar for patients with and without valvular heart disease, including those with MR. Specifically, the rate of stroke and systemic embolism per 100 patient-years in patients with valvular heart disease treated with VKAs was 1.56, while it was 0.83 for those treated with NOACs in the ROCKET-AF trial [35, 36]. Connolly et al. (2022) enrolled 4531 patients with rheumatic heart disease-associated atrial fibrillation to compare the efficacy of non-vitamin K antagonist oral anticoagulants (NOACs), specifically rivaroxaban, with vitamin K antagonists (VKAs). The study found that VKA therapy led to a lower rate of a composite of cardiovascular events or death compared to rivaroxaban, without a higher rate of major bleeding. Additionally, VKA therapy resulted in a lower rate of ischemic stroke and lower mortality due to vascular causes. These findings support current guidelines recommending VKA therapy for stroke prevention in patients with rheumatic heart disease and atrial fibrillation [37]. Consequently, NOACs are recommended over VKAs in patients with AF and MR. In terms of efficacy and safety, Melgaard et al. found that NOACs were associated with a significantly higher risk of thromboembolism compared to warfarin in AF patients with AS, but NOACs were associated with a significantly lower risk of major bleeding compared to warfarin in the same patient group. Furthermore, according to the guidelines from the American College of Cardiology (ACC) and the American Heart Association (AHA), NOACs are not recommended for AF patients with moderate to severe MS due to the absence of supporting evidence in the literature [38].

A number of studies have looked at the effectiveness and safety of various anticoagulants in atrial fibrillation patients in relation to bleeding [34]. In order to lessen the likelihood of bleeding and systemic embolism, this research illuminate the best ways to manage this group of patients. Dabigatran reduced the risk of bleeding in patients with non-valvular AF (NVAF) in the RE-LY trial when compared to other NOACs, including rivaroxaban and apixaban. Patients with NVAF who are more likely to experience bleeding should be evaluated for the possibility of using dabigatran as a therapeutic option. However, each patient’s specific circumstances and considerations should be considered before selecting an anticoagulant [39–41].

Continuing from the previous point, it is crucial to think about the effects of ischemic stroke and MI when managing AF [42]. Ischemic stroke is a major concern for patients with atrial fibrillation due to the higher frequency of thromboembolic events [43]. There is evidence that NOACs are superior to warfarin in preventing ischemic stroke and systemic embolism in patients with atrial fibrillation and native VHD. Apixaban, Dabigatran, and Edoxaban are NOACs that have been shown to decrease hemorrhagic events; however, Rivaroxaban has been associated with an alarming increase in significant bleeding episodes. It is crucial to consider the patient’s risk profile and bleeding tendency before choosing an anticoagulant [44].

Preventing myocardial infarction (MI) is another important therapeutic concern in AF treatment [45]. Treatment decisions should take into consideration the risk of myocardial infarction (MI) and its effects, even if the primary objective of anticoagulant medication is to prevent thromboembolic events. It is important to closely monitor and alter dosages of warfarin as previous research has shown that its effects might vary and that it can interact with other drugs and foods. In contrast, NOACs have the potential to improve patient adherence and treatment results through their streamlined dose regimens and reduced requirement for anticoagulation monitoring [46].

Treatment choices are further complicated when patients with AF undergoing percutaneous coronary intervention (PCI) must also decide on antithrombotic medication [47]. When compared to warfarin-based regimens, the bleeding risk and effectiveness of NOACs combined with single or double antiplatelet therapy are equal, suggesting that they are not inferior. When choosing an antithrombotic medication for atrial fibrillation patients having a percutaneous coronary intervention (PCI) at the same time, it is important to weigh the risks of stent thrombosis, stroke, and bleeding complications [48, 49].

Compliance with NOAC doses and the efficacy of AF medication may interact with one another. Dose once daily has been linked to better patient adherence without sacrificing effectiveness or safety, whereas twice-day dose regimens may provide a more stable risk–benefit profile for stroke prevention. In addition, the patient’s preferences, lifestyle characteristics, renal function, and bleeding risk should be considered when deciding between NOACs and VKAs [50, 51].

Overall, AF therapy necessitates a holistic strategy that harmonizes thromboembolic event avoidance with bleeding and other adverse outcome risk assessment [52]. Anticoagulant therapy, particularly NOACs, has revolutionized the treatment of AF by offering improved safety, efficacy, and convenience compared to traditional VKA therapy. However, the selection of the most appropriate anticoagulant should be guided by careful consideration of individual patient characteristics and preferences, as well as the presence of concomitant conditions such as ischemic stroke and myocardial infarction. By tailoring treatment strategies to the specific needs of each patient, healthcare providers can optimize outcomes and enhance the quality of care for individuals with AF [51].

## Conclusions

The findings of this study indicate that both NOACs and VKAs exhibit comparable efficacy in preventing ischemic stroke in AF patients, regardless of the presence of VHD or N-VHD. Both treatment modalities also demonstrate similar effectiveness in reducing the incidence of myocardial infarction in these patient populations. Importantly, NOACs significantly reduce the risk of intracranial bleeding in both VHD and N-VHD patients. In addition, NOACs are associated with a decreased risk of gastrointestinal bleeding specifically in VHD patients, although this effect does not reach statistical significance in the N-VHD subgroup. Moreover, NOACs are linked to a lower risk of minor and non-fatal bleeding in AF patients with either VHD or N-VHD. These results suggest that NOACs offer a favorable safety profile compared to VKAs, particularly in terms of bleeding complications. However, it is crucial to interpret these findings in the context of current clinical guidelines and to consider potential variations in treatment recommendations for different patient populations.

### Limitation

The included studies presented heterogeneity in the duration of the treatment given to patients. Moreover, in some studies, with a rather short follow-up period, long-term outcomes may not be captured. Furthermore, mitral stenosis (MS) was absent in 50% of this research, and only about 1.6% of the total patient number in the review had MS. This significant underrepresentation of patients with MS is not clearly mentioned or discussed as a limitation. In short, although NOACs appear to be a safer choice over VKAs, especially for bleeding risks, further research with a more homogeneous study population and longer follow-up is needed to confirm these findings and guide clinical guidelines.

## Data Availability

Data available within the article. The authors confirm that the data supporting the findings of this study are available within the article.

## Abbreviations

AF: Atrial fibrillation
CI: Confidence interval
DAPT: Dual antiplatelet therapy
DAT: Dual antithrombotic therapy
ECG: Electrocardiogram
MI: Myocardial infarction
NOS: Newcastle–Ottawa Scale
NOAC: Non-vitamin K oral anticoagulant
NVAF: Non-valvular atrial fibrillation
PCI: Percutaneous coronary intervention
PRISMA: Preferred Reporting Items for Systematic Review and Meta-Analysis
PROSPERO: International Prospective Register of Systematic Reviews
RCT: Randomized controlled trial
RR: Risk ratio
SAPT: Single antiplatelet therapy
SEE: Systemic embolic event
TAT: Triple antithrombotic therapy
VHD: Valvular heart disease
VKA: Vitamin K antagonist
